# Willow Bark (*Salix* spp.) Used for Pain Relief in Arthritis: A Meta-Analysis of Randomized Controlled Trials

**DOI:** 10.3390/life13102058

**Published:** 2023-10-14

**Authors:** Chun-Ru Lin, Sung Huang Laurent Tsai, Che Wang, Cheng-Lin Lee, Shao-Wen Hung, Yi-Tang Ting, Yu Chiang Hung

**Affiliations:** 1 Department of Medical Education, Chang Gung Memorial Hospital, Linkou Branch, No. 5, Fuxing Street, Guishan District, Taoyuan City 333, Taiwan; 2 Department of Orthopaedic Surgery, Chang Gung Memorial Hospital, Keelung Branch, No. 222, Maijin Rd., Anle Dist., Keelung City 204006, Taiwan; 3 Department of General Medicine, Chi Mei Medical Center, No. 901, Zhonghua Rd., Yongkang District, Tainan City 71004, Taiwan; 4 Department of Pediatrics, Chang Gung Memorial Hospital, Linkou Branch, No. 5, Fuxing Street, Guishan District, Taoyuan City 333, Taiwan; 5 Department of Medical Education, Taipei Municipal Wanfang Hospital, No. 111, Sec. 3, Xinglong Rd., Wenshan District, Taipei City 116, Taiwan; 6 Department of Chinese Medicine, Kaohsiung Chang Gung Memorial Hospital, No. 123, Dapi Road, Niaosong District, Kaohsiung City 833401, Taiwan

**Keywords:** arthritis, osteoarthritis, rheumatoid arthritis, willow bark, herbal medicine, pain, adverse events

## Abstract

This study intends to assess the analgesic effects, physical facilitation, and safety of willow bark use in patients with arthritis. Our study was conducted based on the Preferred Reporting Items for Systematic Reviews and Meta-Analyses statement. PubMed, Scopus, EMBASE, Web of Science, Cochrane, and ClinicalTrials.gov were searched for relative randomized controlled trials (RCTs) describing the efficacy or adverse events of willow bark in patients with arthritis until 12 April 2023. We used Cochrane ROB 2.0 and the Grading of Recommendations, Assessment, Development, and Evaluations system to evaluate the quality of studies and evidence. The meta-analysis was carried out by the fix-effects model. This study included five studies with six RCTs consisting of 329 patients with arthritis. The results showed significant differences in pain relief and improvement in physical status for patients with arthritis between willow bark treatment and placebo groups, and no significant differences in the risk of all adverse events in patients with arthritis between willow bark treatment and placebo. Owing to the potential bias, the certainty and evidence of our findings are still inadequate. Therefore, further RCTs are needed to confirm our results.

## 1. Introduction

Arthritis, derived from the old Greek term “diseases of the joints”, refers to inflammation with co-existed pain or structural damage. There are more than 100 types of arthritis, characterized by pain, stiffness, swelling, loss of function, weakness, deformity, and instability of joints. Both osteoarthritis and rheumatoid arthritis are common types of arthritis [1]. The affected joints can be found in the knees, hips, shoulders, and hands. Different joints are often susceptible to different arthritis. The treatment strategies also vary. General treatments for osteoarthritis include exercises, physical therapy, topical or oral medications for pain control [2], and surgical intervention for joint replacement [3]. As for rheumatoid arthritis, anti-inflammatories are used for early disease remission and preventing radiographic progression [4]. In the United States, approximately 22.7% of adults have received a diagnosis of arthritis, with 43.5% of those individuals experiencing activity limitations as a result of the condition [5]. The average medical cost for an individual with arthritis was estimated at USD 9554 annually and patients with arthritis resulted in USD 460 billion in all-cause medical costs [6]. The high prevalence of arthritis takes a toll on the economy and the patient’s life quality. Therefore, exploring effective and economical ways of treatment becomes a pressing issue.

Willow bark, also known as *Salix*, has been historically used for medicinal purposes for over 3500 years [7]. In the ancient world, people in Egypt, South America, Classical Greece, and China used willow bark as medicine. Sumerians and Ancient Egyptians utilized it as a painkiller and antipyretic. The therapeutic benefits of willow bark were recognized by ancient Roman and Greek physicians, including Hippocrates. In the fourth century BC, Hippocrates used willow bark to treat inflammatory pain [8]. Over time, the use of willow bark became more widespread. In 1763, Reverend Edward Stone conducted the first clinical research study on willow bark, confirming its antipyretic effects [7]. In 1827, salicylic acid, which was the active component of willow bark, was extracted and isolated by Johann Andreas Buchner [9]. In 1853, Charles Gerhardt manufactured acetylsalicylic acid [10]. In 1869, the structure of acetylsalicylic acid was accurately reported [11]. In 1897, salicin was successfully refined into aspirin [7,12]. In 1987, the chemists of Bayer synthesized a steady acetylated salicylate from salicylic acid. Salicylic acid was named aspirin by combining the two words acetyl and Spirsäure [13].

Aspirin could inhibit cyclooxygenase (COX), which inhibited prostaglandin synthesis and resulted in antipyretic, analgesic, and anti-inflammatory effects [14]. Even though salicin in willow bark served as a precursor to aspirin, its medical effect could not be solely attributed to it. Willow bark extract is commonly employed as a complementary therapy for pain and inflammation management, such as those related to low back pain, osteoarthritis, tendinitis, bursitis, and headaches. Nonetheless, the safety and effectiveness of willow bark extract are still being scrutinized [15].

There are several mechanisms to explain the adverse impact of arthritis on individuals. Osteoarthritis, the most prevalent arthritis, is characterized by the degeneration of cartilage. Cartilage degeneration can be triggered by excessive body weight, joint injuries, or advanced age. The characteristics of osteoarthritis are the remodeling of subchondral bone, the formation of ectopic bone and osteophytes, the hypertrophy of the joint capsule, and the inflammation of the synovial lining [16]. Of the individuals with osteoarthritis, 25% cannot perform major activities of daily living, and 80% suffer from some movement limitation [17]. With reduced physical activity caused by osteoarthritis, patients experience 20% higher age-adjusted mortality [18]. The common medication for osteoarthritis is topical or oral non-steroidal anti-inflammatory drugs (NSAIDs), intra-articular steroid injections, and duloxetine [18]. The toxicity of NSAIDs includes decreasing renal blood flow, gastrointestinal ulceration, and bleeding. To prevent the gastrointestinal ulceration caused by NSAIDs, misoprostol, proton pump inhibitors, and histamine-2-receptor antagonists were helpful [19]. The side effects of intra-articular steroid injections included the destruction of cartilage, infectious arthritis, and the systemic effects of steroids [20]. The major adverse events of duloxetine for osteoarthritis were nausea, constipation, and fatigue [21]. As for rheumatoid arthritis, a chronic systemic autoimmune disease, the inflammation of the joint is induced by a reaction of autoantibody to self-citrullinated protein [22]. Joint inflammation and joint deformity impairments in rheumatoid arthritis then contribute to functional limitations in daily life [23]. As for rheumatoid arthritis, first-line treatments aim to control pain and reduce inflammation, so analgesics such as NSAIDs and corticosteroids are often used. Second-line treatment is disease-modifying antirheumatic drugs (DMARDs), promoting joint destruction and deformity remission. DMARDs include methotrexate, hydroxychloroquine, and sulfasalazine [24]. The adverse events of systemic corticosteroid therapy for rheumatoid arthritis included hyperglycemia, immunosuppression, gastrointestinal events, and osteoporosis [25]. Of all the DMARDs, hydroxychloroquine was the safest drug. Hydroxychloroquine did not increase the risk of hepatotoxicity, severe infection, and renal dysfunction. The major side effects of hydroxychloroquine included diarrhea and rash. Methotrexate, sulfasalazine, and leflunomide have similar side effects, including bone marrow suppression, allergy, gastrointestinal events, and severe infections [26].

Multiple animal models and in vitro trials possibly explained the mechanisms of efficacy from the willow bark extract. For instance, willow bark extract inhibits pro-inflammatory cytokines, such as tumor necrosis factor α (TNFα), cyclooxygenase-2 (COX-2), and the nuclear translocation of the transcription factor in proinflammatory activated monocytes, resulting in its anti-inflammatory effect [27]. Moreover, willow bark extract has the therapeutic effect of preventing oxidative stress [28] and induces apoptosis in human colon and lung cancer cells [29]. Several studies have been conducted to analyze which component in the willow bark is responsible for its therapeutic effect. There were at least 13 different main compounds, including saligenin, salicylic acid, salicin, isosalicin, picein, salidroside, triandrin, salicoylsalicin, salicortin, isosalipurposide, salipurposide, naringenin-7-O-glucoside, and tremulacin, in willow bark identified and analyzed with the high-performance liquid chromatography technique and mass spectrometry [30]. As the precursor of aspirin, however, salicin cannot fully explain the clinical effect of willow bark [31]. Both flavonoids and polyphenols in the willow bark were proven to be attributed to anti-inflammatory effects [32]. Willow bark extract is now widely used for conditions associated with inflammation or fever, and it can be applied to various types of pain, such as joint or knee pain, acute back pain, osteoarthritis, headache, menstrual cramps, tendonitis, and generalized pain [33]. For arthritis per se, the efficacy of willow bark from various studies is diverse. Three studies with RCT showed that both willow bark extract alone [34] and compound drugs [35,36] had an analgesic effect versus placebo. In contrast, three studies with RCT showed that willow bark extract yielded no significant benefit [37,38]. According to Biegert et al., although salicin derivatives in the willow bark were metabolized in vivo to salicylic acid, serum salicylate concentration was too low to reach clinical effects [37]. In addition, the inhibition mechanism of the COX-2-mediated release of prostaglandin E2 was confirmed in vitro, but still short of proof of in vivo trials [39].

Prior research reported that willow bark may improve physical function and relieve pain in patients with joint disorders [34]. However, there is no systematic review to assess the efficacy and safety of willow bark in patients with arthritis. Thus, we conducted a meta-analysis of the relevant literature to evaluate the pain relief, improvement of physical function, and occurrence of adverse events associated with the use of willow bark in patients with arthritis.

## 2. Materials and Methods

### 2.1. Research Protocol and Research Question

The study was conducted following the guidelines of the Preferred Reporting Items for Systematic Reviews and Meta-Analyses (PRISMA) statement. The protocol of this systematic review and meta-analysis study has been submitted in PROSPERO (CRD42023417496). We formulated our research question to focus on patients with arthritis, investigating the use of willow bark (*Salix* spp.) versus placebo in managing pain and adverse events.

### 2.2. Eligibility Criteria and Primary Outcome

We included studies according to the following criteria: (1) the study must include patients with arthritis, (2) the study design must be a randomized controlled study (RCT), and (3) the study outcomes must include pain scores (e.g., Visual Analogue Scale (VAS), Numerical Rating Scale (NRS), or Brief Pain Inventory (BPI)), the Western Ontario and McMaster Universities Osteoarthritis Index (WOMAC), or adverse events. We excluded the studies if they were (1) single-arm follow-up studies, (2) case series, case reports, basic science experiments, reviews, or non-human studies, (3) conference abstracts, and (4) non-English articles.

### 2.3. Search Strategy and Study Selection

On 12 April 2023, a systematic search was conducted across multiple databases, including PubMed, Scopus, EMBASE, Web of Science, The Cochrane Library, and ClinicalTrials.gov. The search utilized a combination of relevant keywords (e.g., willow bark, arthritis, pain, etc.). In addition, the reference lists of the included studies were screened by two independent reviewers to ensure a comprehensive search. Two independent reviewers (CRL, CW) evaluated the eligibility of the articles based on their titles and abstracts. The same reviewers then evaluated full-text articles to make the final inclusion decisions. Any conflicts of opinion between the reviewers were resolved through discussion.

### 2.4. Data Collection and Quality Assessment

Three independent reviewers (CRL, CW, CLL) extracted relevant data from the included studies. The extracted data comprised study characteristics such as author, year of publication, region of study, data source, study design, and period of study. The sample size, patient age, inclusion criteria for each study, and specific definitions of each treatment arm were also recorded. Additionally, outcomes of interest such as pain scales, physical function scores, and adverse events were documented. Two reviewers (CRL, SHLT) evaluated the risk of bias in the included studies and the quality of evidence in the study outcomes using Cochrane ROB 2.0 [40] and the GRADE (Grading of Recommendations, Assessment, Development, and Evaluations) systems, respectively [41]. Any discrepancies among the reviewers were solved through discussion.

### 2.5. Statistical Analysis and Quantitative Data Synthesis

We conducted a pairwise meta-analysis to compare the efficacy and safety of willow bark use in patients with arthritis. To evaluate the analgesic effect and improvement of the joint function of willow bark, standardized mean differences (SMD) were used. In addition, we calculated odd ratios (ORs) for the risk of adverse events of willow bark use in patients with arthritis. Moreover, the risk of adverse events was calculated by dividing the number of patients experiencing adverse events by the number of total patients in each trial. We assessed the statistical heterogeneity of the results by defining I^2^ values of 25–50%, 51–75%, and 76–100% as low, moderate, and high statistical heterogeneity, respectively [42]. Due to the expected clinical heterogeneity of the included studies, we used the fixed-effects model to estimate the pooled results in this meta-analysis. A *p*-value less than 0.05 was considered statistically significant for all analyses.

## 3. Results

### 3.1. Literature Search and Selection Process

**Figure 1:**
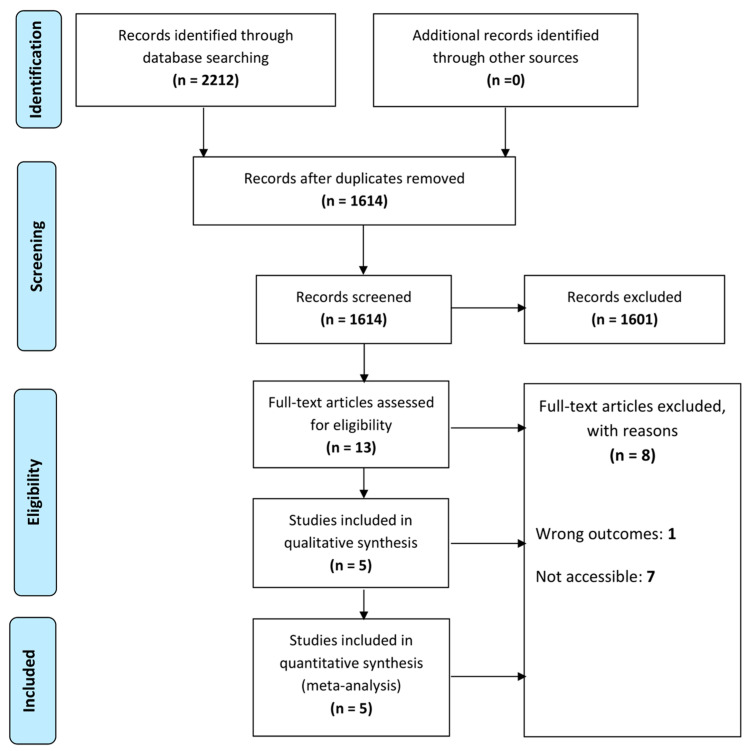
Flow of identification, screening, eligibility, and inclusion.

A comprehensive search across various electronic databases yielded 2212 records. After eliminating duplicate and irrelevant studies by scrutinizing the titles and abstracts, 13 full-text articles were assessed for eligibility. Finally, five studies with six RCTs comprising a total of 329 participants were included in this meta-analysis (Figure 1).

### 3.2. Study Characteristics

Table 1 reports the study characteristics of the included studies. There were two studies conducted in Germany (*n* = 188) [34,37], one study conducted in the United States (*n* = 100) [35], one study conducted in the United Kingdom (*n* = 14) [38], and one study conducted in Canada (*n* = 27) [36]. All studies used oral willow bark and were published in full articles. There were two studies that used willow bark extract [34,37]. Three studies used compound drugs containing willow bark [35,36,38]. There was one study containing two RCTs that involved participants with osteoarthritis and rheumatoid arthritis [37]. Table 2 and Table 3 reported the information on the formulation, compound, and concentration used in the included studies.

### 3.3. Methodological Quality and Assessment of Risk of Bias

**Figure 2:**
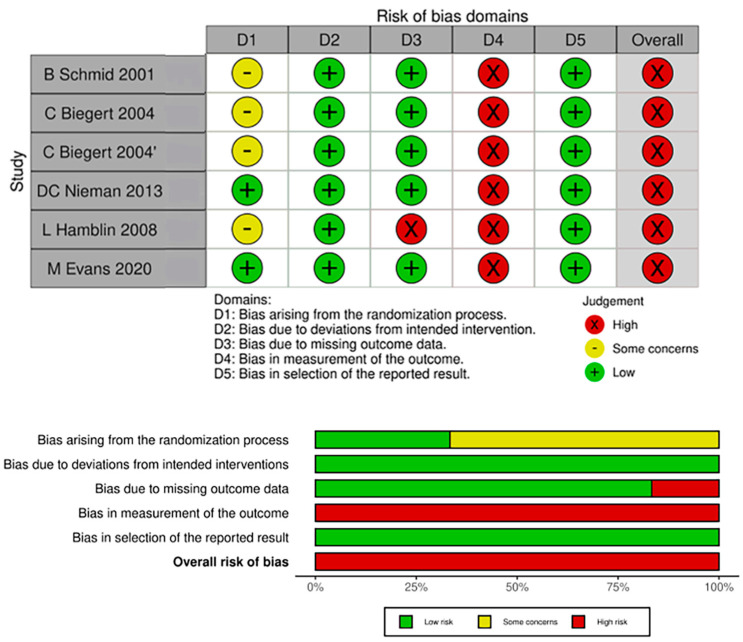
ROB2, risk of bias assessment of the included studies, and the summary of domains. The study conducted by C. Biegert et al. comprised two distinct trials. The first trial focused on osteoarthritis and was designated as “C Biegert 2004”. The second trial, which centered on rheumatoid arthritis, was labeled “C Biegert 2004′”. See refs. [34,35,36,37,38].

According to ROB 2.0, five studies with six RCTs were rated as high risk of bias (Figure 2). In the risk of bias due to the random sequence generation, baseline data in three RCTs had differences [34,37], and one RCT did not report baseline balance characteristics [38], which was rated as some concern. In the risk of bias resulting from missing outcome data, one study did not explain the reasons why the participants lost follow-up and were rated as high risk of bias [38]. In the risk of bias owing to the measurement of the outcome, all six RCTs used subjective patient-reported outcome scales, which were rated as high risk of bias [34,35,36,37,38]. Table 4 summarizes the outcome of GRADE of the included studies.

### 3.4. Pain

**Figure 3:**
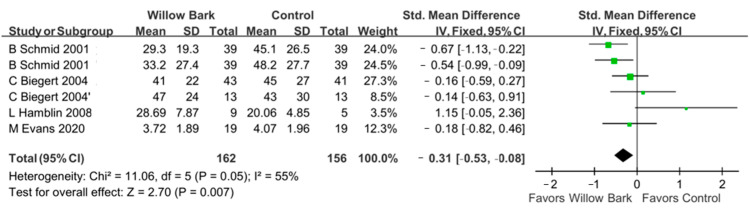
Forest plot demonstrating overall pain scores comparing willow bark and placebo. Better pain control is shown in favor of willow bark over placebo. The study conducted by C. Biegert et al. comprised two distinct trials. The first trial focused on osteoarthritis and was designated as “C Biegert 2004”. The second trial, which centered on rheumatoid arthritis, was labeled “C Biegert 2004′”. See refs. [34,36,37,38].

There were four studies with five RCTs involving 229 patients with arthritis included to evaluate the analgesic efficacy of willow bark [34,36,37,38]. Figure 3 reports the difference in analgesic effect between the willow bark and placebo groups. Our meta-analysis showed a statistically significant difference in pain controls for patients with arthritis between the willow bark and placebo groups (SMD: −0.31; 95% CI: −0.53, −0.08, *p* = 0.007; I^2^: 55%; quality of evidence: very low).

### 3.5. WOMAC

**Figure 4:**
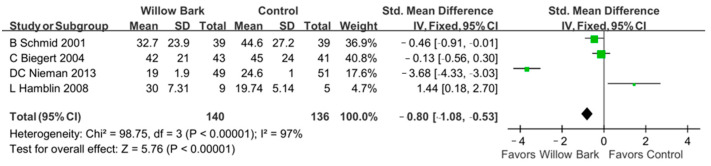
Forest plot demonstrating overall WOMAC scores comparing willow bark and placebo. A better effect of physical improvement is shown in favor of willow bark over placebo. See refs. [34,35,37,38].

There were four studies with four RCTs involving 276 patients with arthritis included to evaluate the efficacy of willow bark in improving health status [34,35,37,38]. Figure 4 reports the difference in WOMAC scores between the willow bark and placebo groups. Our meta-analysis showed a statistically significant difference in the improvement of health status for patients with arthritis between the willow bark and placebo groups (SMD: −0.80; 95% CI: −1.08, −0.53, *p* < 0.001; I^2^: 97%; quality of evidence: very low).

### 3.6. Adverse Event

**Figure 5:**
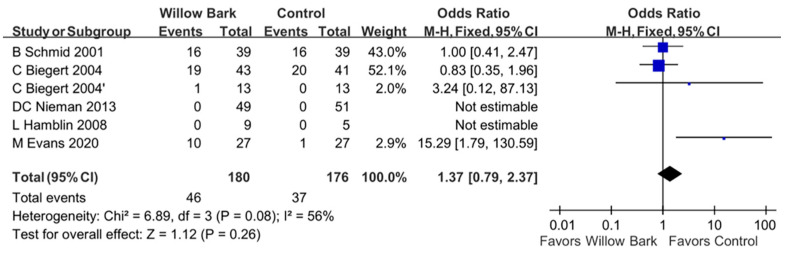
Forest plot demonstrating overall adverse events between willow bark and placebo. A lower adverse event is shown in favor of willow bark over placebo. The study conducted by C. Biegert et al. comprised two distinct trials. The first trial focused on osteoarthritis and was designated as “C Biegert 2004”. The second trial, which centered on rheumatoid arthritis, was labeled “C Biegert 2004′”. See refs. [34,35,36,37,38].

There were five studies with six RCTs involving 329 patients with arthritis included to evaluate the risk of adverse events of willow bark [34,35,36,37,38]. Figure 5 reports the different risks of adverse events between the willow bark and placebo groups. In our study, we found no significant difference in the risk of all adverse events in patients with arthritis treated with willow bark compared with analgesics or placebo in our meta-analysis (odds ratio, OR: 1.37; 95% CI: 0.79, 2.37, *p* = 0.26; I^2^: 56%; quality of evidence: very low). Table 5 summarizes the adverse events reported in the included studies.

### 3.7. Post Hoc Analysis

**Figure 6:**
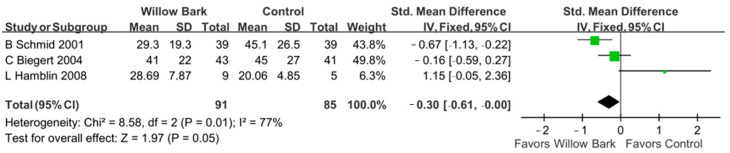
Post hoc analysis of only studies including WOMAC pain scores comparing willow bark and placebo. Better pain control is shown in favor of willow bark over placebo. See refs. [34,37,38].

**Figure 7:**
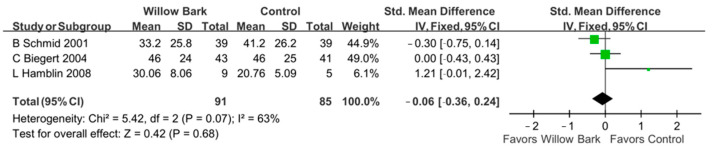
Post hoc analysis of only studies including WOMAC stiffness scores comparing willow bark and placebo. A better effect of stiffness relief is shown in favor of willow bark over placebo. See refs. [34,37,38].

**Figure 8:**
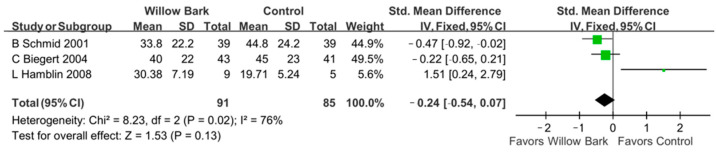
Post hoc analysis of only studies including WOMAC physical function scores comparing willow bark and placebo. A better effect of physical improvement is shown in favor of willow bark over placebo. See refs. [34,37,38].

In the post hoc analysis, three subgroups of the WOMAC score were analyzed, including the WOMAC pain score, the WOMAC stiffness score, and the WOMAC physical function score. There are three studies with three RCTs reporting the aforementioned outcomes [34,37,38]. In the group of WOMAC pain score, Figure 6 reports that there was a significant difference in pain relief between willow bark and analgesic drugs or placebo for patients with arthritis (SMD: −0.30; 95% CI: −0.61, 0.00, *p* = 0.05; I^2^: 77%; quality of evidence: very low). In the group of WOMAC stiffness score, Figure 7 reports that there were no significant differences in symptom relief between willow bark and analgesic drugs or placebo for patients with arthritis (SMD: −0.06; 95% CI: −0.36, 0.24, *p* = 0.68; I^2^: 63%; quality of evidence: very low). In the group of WOMAC physical function score, Figure 8 reports that there were no significant differences in physical function improvement between willow bark and either analgesic drugs or placebo for patients with arthritis (SMD: −0.24; 95%, CI: −0.54, 0.07, *p* = 0.13; I^2^: 76%; quality of evidence: very low).

## 4. Discussion

Our study revealed the potential clinical benefits of willow bark for pain relief and the improvement of physical function in patients with arthritis. In addition, there were no significant differences in the risk of adverse events among patients with arthritis between the willow bark and analgesics or placebo groups. These adverse events mainly consisted of skin and appendage disorders, gastrointestinal system disorders, and infections associated with using willow bark.

In our study, the most common adverse event of willow bark was gastrointestinal system disorders (*n* = 14) and central and peripheral nervous system disorders (*n* = 14) followed by skin and appendage disorders (*n* = 9). However, no severe adverse event was noted. In our study, there were no significant differences in the risk of adverse events in arthritis patients between using willow bark and analgesics or placebo groups. Previous review articles reported that the most common adverse events of willow bark were gastrointestinal system disorders, including upper abdominal pain, nausea, gastric disorder, and dyspepsia [13], and this result was compatible with our findings. The potential toxicity and adverse effects of willow bark extraction included anaphylactic reaction, gastrointestinal upset, and a remarkable capacity to concentrate heavy metals, especially cadmium [43]. Components in willow bark may interact with anticoagulants (increase bleeding tendency), beta-blockers and diuretics (decrease the effect of the drugs), and NSAIDs (increase the risk of stomach bleeding) [33]. Only a limited number of case reports indicated serious adverse events including anaphylactic reaction [44] and acute respiratory distress syndrome [45]. The use of willow bark as a medicine should be carefully assessed for those with a history of allergy to salicylates. Additionally, children under the age of 16 were suggested to avoid using willow bark with the concern of Reye syndrome [33].

Our study showed potential benefits in treating arthritis with willow bark. Nonetheless, some limitations were present in our study. Only five studies with six RCTs qualified for meta-analysis, so the sample size was relatively small. In addition, there was no standard regimen of willow bark for arthritis. The designs of these trials were also different. Some studies used willow bark extract alone as medication, the others used compound drugs with other components, which might have an impact on its analgesic effect. There were three studies containing other compounds because the authors designed these studies and thought it was a complete regimen for therapeutic effect. Since willow bark was still the primary substance in the regimen, and other compounds were regarded as adjuvant, we still included these three studies in the analysis. However, potential analgesic effects may be presented in other botanical substances. Clinical heterogeneity should be also noted when interpreting our study results. To solve this condition, we used the GRADE system in our analysis. In addition, all six RCTs used subjective patient-reported outcome scales. Both WOMAC and pain scores (e.g., VAS, NRS, or BPI) were not objective. Although there were many studies to verify the validity and reliability of WOMAC, it still had limitations [46], especially for the convergent validity of the stiffness subscale [47]. To solve this problem, a post hoc analysis was conducted in this study. This is the first meta-analysis of RCTs to assess the clinical advantages of willow bark in diminishing pain and enhancing physical function in patients with arthritis. Willow bark may be one of the alternative treatments for knee osteoarthritis. To confirm our findings, additional RCTs with willow bark extract and standard regimens are warranted in the future.

## 5. Conclusions

Our meta-analysis and systematic review reported that there were significant differences in pain reduction and improvement of health status among patients with arthritis between the willow bark and placebo groups. In addition, no significant differences were revealed in the risk of adverse events among patients with arthritis between the willow bark and placebo groups. Owing to the potential bias, small sample sizes, and inconsistencies in these included studies, the certainty and evidence of our findings are still inadequate. Therefore, further RCTs are needed to confirm our results.

## Figures and Tables

**Table 1:** Study characteristics of the included studies.

Study	Design	Location	Drug Type	Inclusion Criteria	Experimental Group	Control Group	Age (Years)	Sex (M/F)	Outcome	Follow-Up
B Schmid 2001 [34]	RCT	Germany	Oral	Osteoarthritis of the hip or the knee	Willow bark extract two tablets twice daily for two weeks (corresponding to a dose of 240 mg salicin/day), 39 participants	Placebo two tablets twice daily for two weeks, 39 participants	Willow bark group: 52.4 ± 7.0, placebo group: 53.5 ± 10.5	59/19	Lequesne index, WOMAC-VA 3.0, diary VAS	14 days
C Biegert 2004 [37]	RCT	Germany	Oral	Osteoarthritis of the hip or knee	Willow bark extract two tablets twice daily for six weeks (corresponding to a dose of 240 mg salicin/day), 43 participants	Placebo two tablets twice daily for six weeks, 41 participants	Willow bark 62.9 ± 7.2, diclofenac 61.2 ± 6.6, placebo 62.4 ± 8.9	43/41	WOMAC-VA 3.0, overall efficacy, quality of life assessment (SF-36), tolerability (100 mm VAS)	6 weeks
C Biegert 2004′ [37]	RCT	Germany	Oral	Rheumatoid arthritis	Willow bark extract two tablets twice daily for six weeks (corresponding to a dose of 240 mg salicin/day), 13 participants	Placebo two tablets twice daily for six weeks, 13 participants	Willow bark 56.5 ± 8.9, placebo 60.1 ± 11.0	4/22	Pain score (VAS), number of tender/painful and swollen joints, physical function (HAQ disability index), severity of morning stiffness, overall efficacy, quality of life (SF-36 index), ESR, CRP in plasma concentration, numbers of patient had ACR criteria for improvement	6 weeks
DC Nieman 2013 [35]	RCT	United States	Oral	Joint pain in the knees, hip, ankles, shoulders, or hands	Instaflex™ Joint Support three capsules per day for eight weeks, 49 participants	Placebo (magnesium stearate), three capsules per day, 51 participants	Instaflex group: 57.6 ± 0.9, placebo group: 58.3 ± 0.8	17/83	WOMAC, health-related quality of life (SF-36), symptom logs, joint pain (12-VS), 6 min walk test, blood measure (CRP, IL-1, TNFα, IL-8, IL-10)	8 weeks

**Table 2:** Patented formulations and botanical or chemical medications from the included studies.

Study	Formulation	Source	Species, Concentration	Quality Control Reported? (Y/N)	Chemical Analysis Reported? (Y/N)
DC Nieman 2013 [35]	Instaflex™ contains: glucosamine sulfate (1500 mg) methylsulfonyl-methane (MSM) (500 mg) white willow bark extract (standardized to 15% salicin) (250 mg) ginger root concentrate (50 mg) Boswellia serrata extract (standardized to 65% boswellic acid) (125 mg) turmeric root extract (50 mg) cayenne 40 m H.U. (50 mg) hyaluronic acid (4.0 mg)	Direct Digital, Charlotte, United State, North Carolina,	white willow bark, 15% salicin (250 mg) Boswellia serrata, 65% boswellic acid (125 mg)	N	Y
M Evans 2020 [36]	Pain Bloc-R contains (per capsule): Vitamin D3 (as cholecalciferol) (500 IU) Vitamin B12 (0.5 mg) White willow bark extract (std. to 15% salicin) (*Salix alba*) (150 mg) Angelica root (Angelica dahurica) (50 mg) Acetyl L-carnitine HCl (50 mg) Caffeine (from Green Coffee bean, Coffea arabica) (37.5 mg) L-Theanine (37.5 mg) BenfoPure Benfotiamine (25 mg) Pyridoxal 5 Phosphate (17 mg) L-Tetrahydropalmatine (25 mg)	LifeSeasons Inc., United Stated, Texas	White willow bark extract (std. to 15% salicin) (*Salix alba*) (150 mg) Angelica root (Angelica dahurica) (50 mg)	N	Y

**Table 3:** Isolated chemical compounds from the included studies.

Study	Compound, Concentration	Source	Purity (%) (and Grade, If Applicable)	Quality Control Reported? (Y/N)
B Schmid 2001 [34]	Salicin, 17.6%	*Salix purpurea* × *daphnoides*	N	N
C Biegert 2004 [37]	Salicin, 15%	*Salix daphnoides*	N	N
L Hamblin 2008 [38]	Nil	White willow, wild yam, bogbean, meadowsweet, black cohosh, ginger, dandelion root, celery seed, licorice, devil’s claw, turmeric (did not report the concentration of every herb)	N	N

**Table 4:** GRADE (Grading of Recommendations, Assessment, Development, and Evaluations) criteria for assessing the quality of evidence.

Outcome	Number of Studies	Number of Participants	Risk of Bias	Imprecision	Inconsistency	Indirectness	Publication Bias	Relative Effect (95% Confidence Interval)	Confidence in Effect Estimate (Grade)
Pain	4	229	Serious	Serious	Moderate	Not serious	Serious	−0.31 (−0.53, −0.08)	Very low
WOMAC	4	276	Serious	Serious	Serious	Not serious	Serious	−0.80 (−1.08, −0.53)	Very low
Adverse effect	5	329	Serious	Serious	Not serious	Not serious	Serious	1.37 (0.79, 2.37)	Very low

**Table 5:** Adverse events comparing willow bark and placebo.

	Willow Bark	Placebo
Skin and appendage disorders	9	7
GI system disorders	14	26
Central and peripheral nervous system disorders	14	19
General disorders	7	4
Psychiatric	1	0
Cardiovascular disorder	1	1
Urinary system disorder	1	2
Vision disorders	1	0
Vascular disorders	0	1
Infections	2	2
Change in hemogram	2	2
Hypertriglyceridemia	1	1
Musculoskeletal system disorders	4	5
Disc prolapse	1	0
Other adverse events	3	5

## Data Availability

All the data in this study are reported in the article. For further information, please contact the corresponding author.
